# Nonlinear 2-DOF PID controller optimized by artificial lemming algorithm for robust engine speed regulation in spark-ignition systems

**DOI:** 10.1038/s41598-025-27873-2

**Published:** 2025-11-26

**Authors:** Serdar Ekinci, Davut Izci, Mostafa Jabari, Mohit Bajaj, Vojtech Blazek, Lukas Prokop

**Affiliations:** 1 Department of Computer Engineering, Bitlis Eren University, Bitlis, 13100, Turkey; 2 Department of Electrical and Electronic Engineering, Bursa Uludag University, Bursa, 16059 Turkey; 3 Applied Science Research Center, Applied Science Private University, Amman, 11931 Jordan; 4 Faculty of Electrical Engineering, Sahand University of Technology, Tabriz, Iran; 5 Department of Electrical Engineering, Graphic Era (Deemed to be University), Dehradun, 248002 India; 6 Graphic Era Hill University, Dehradun, 248002 India; 7 ENET Centre, CEET, VSB-Technical University of Ostrava, Ostrava, 708 00 Czech Republic

**Keywords:** Artificial lemming algorithm, Automotive control, Disturbance rejection, Engine speed regulation, Metaheuristic optimization, Nonlinear systems, PID tuning, Two-degree-of-freedom PID, Energy science and technology, Engineering, Mathematics and computing

## Abstract

Achieving precise and stable engine speed regulation in spark-ignition (SI) systems remains a challenging task because of the inherent nonlinearities, time-varying characteristics, and external disturbances of internal combustion engines (ICEs). Conventional proportional–integral–derivative (PID) controllers often fail to simultaneously ensure fast tracking and robust disturbance rejection under dynamic operating conditions. To address this limitation, a nonlinear two-degree-of-freedom (2-DOF) PID controller has been developed and optimized using the artificial lemming algorithm (ALA) which is a recent bio-inspired metaheuristic that mimics lemming population behaviors to balance exploration and exploitation adaptively through an energy-driven mechanism. The proposed controller was implemented on a detailed mathematical model of the SI engine, encompassing throttle dynamics, manifold pressure variation, combustion torque generation, and crankshaft motion. A multi-term cost function combining normalized overshoot, steady-state error, and stability coefficients was minimized to determine optimal controller gains. Extensive experiments were conducted, including statistical robustness evaluation, transient and steady-state analyses, trajectory tracking, and disturbance-rejection tests. ALA exhibited the lowest mean and standard deviation of the cost function (4.7170 and 0.1429, respectively), confirming its strong convergence stability compared to the starfish optimization algorithm, parrot optimizer, coati optimization algorithm, and dwarf mongoose optimizer. The ALA-optimized controller achieved a rise time of 0.3114 s, a settling time of 2.4313 s, an overshoot of only 0.0027%, and an extremely small steady-state error of 2.62 × 10⁻¹¹%. Furthermore, the controller demonstrated superior trajectory-tracking accuracy and exceptional disturbance-rejection capability, maintaining speed deviations below 0.5% under abrupt load torque perturbations. The results confirm that the ALA-based nonlinear 2-DOF PID controller provides a robust and energy-efficient solution for nonlinear engine speed regulation, outperforming recent metaheuristic-based approaches in both accuracy and reliability. Owing to its adaptive and scalable design, the proposed control framework is well-suited for integration into real-time embedded engine control units, hybrid powertrains, and other nonlinear dynamic systems requiring high-precision regulation under uncertainty.

## Introduction

Precise and stable regulation of engine speed in spark-ignition (SI) systems has long been a central issue in automotive control engineering due to the inherently nonlinear, time-varying, and disturbance-prone nature of internal combustion engines (ICEs)^1,2^. The interaction among throttle dynamics, manifold pressure, combustion torque, and mechanical load introduces strong coupling and parameter uncertainty, which frequently leads to degraded speed stability during rapid transients. These challenges are further intensified by variations in ambient conditions, air–fuel ratio fluctuations, and combustion delays. Therefore, maintaining engine speed close to the desired reference while simultaneously minimizing overshoot, oscillations, and steady-state error remains a demanding control objective.

Conventional proportional–integral–derivative (PID) controllers have been the dominant choice in automotive applications because of their straightforward structure, cost-effectiveness, and ease of implementation^3,4^. However, their performance degrades significantly under nonlinear operating conditions or when the engine is subjected to dynamic load disturbances^5–7^. Numerous studies have attempted to enhance PID robustness through modifications such as fractional-order PID (FOPID)^8,9^, two-degree-of-freedom (2-DOF) PID^10,11^, and PIDA extensions^12,13^. Despite these advancements, most traditional tuning strategies rely on analytical approximations or manual adjustments that fail to achieve optimal gain combinations in complex multi-variable environments^14^. Particularly in SI engines, where combustion and mechanical interactions are nonlinear, classical methods often result in slow convergence, excessive oscillations, or limited adaptability to torque disturbances.

The 2-DOF PID controller has recently emerged as an attractive solution because it introduces two independent tuning channels that separately handle setpoint tracking and disturbance rejection^11,15^. This architecture allows fine control of reference tracking without compromising robustness against external perturbations, making it suitable for dynamic systems such as engines and electric drives. Nonetheless, its success depends strongly on the quality of parameter tuning, which presents a high-dimensional, nonlinear, and non-convex optimization problem. Analytical tuning formulas are inadequate in such cases, motivating the use of metaheuristic and bio-inspired optimization algorithms that can efficiently explore complex search spaces and avoid local minima^16–20^.

The integration of metaheuristic algorithms into control design has revolutionized classical tuning approaches. Early works using genetic algorithms (GA) and particle swarm optimization (PSO) demonstrated improved convergence and adaptability but often suffered from premature stagnation and slow response^15^. Subsequent bio-inspired algorithms (including the aquila optimizer (AO)^21,22^, electric eel foraging optimization (EEFO)^1^, and coati optimization algorithm (COA)^16^ have achieved notable improvements in control performance, yet each exhibits specific trade-offs between exploration and exploitation. For instance, the bat algorithm (BA) achieved enhanced regulation in SI engines under load/no-load conditions but was limited by local convergence tendencies^2^. Similarly, the particle-swarm-based 2-DOF PID controller reported in hybrid-vehicle thermal systems improved transient characteristics but lacked robustness under dynamic load variations^10^.

Recent advancements have also focused on hybrid and learning-enhanced metaheuristics that incorporate adaptive mechanisms to maintain search diversity. The starfish optimization algorithm (SFOA)^17^ and parrot optimizer (PO)^18^ have demonstrated strong global exploration in nonlinear system identification and medical applications, while the dwarf mongoose optimizer (DMO)^23^ introduced social hierarchy mechanisms to improve stability and convergence. Despite these improvements, many of these algorithms remain vulnerable to excessive randomness, slow convergence rates, or oscillatory behavior when applied to nonlinear control design^24,25^. Hence, the need persists for a more reliable and dynamically adaptive optimizer capable of balancing exploration and exploitation in a problem-specific manner.

The artificial lemming algorithm (ALA) was recently introduced as a bio-inspired optimization technique that simulates the complex migratory, foraging, and survival behaviors of lemming populations^26^. Unlike most existing methods, ALA incorporates an energy-driven behavioral mechanism that gradually decreases an agent’s energy level over iterations, thereby governing the smooth transition between exploration (global search) and exploitation (local refinement). This adaptive mechanism allows the algorithm to dynamically regulate search intensity and prevent premature convergence, achieving high accuracy and stability across a wide range of benchmark problems. Its demonstrated success in high-dimensional, non-convex optimization tasks suggests strong potential for engineering control applications requiring precision and robustness. Nevertheless, ALA’s capabilities have not yet been systematically applied to tuning advanced controllers in nonlinear dynamic systems such as spark-ignition engines.

This research bridges that gap by employing ALA to optimize a nonlinear two-degree-of-freedom PID controller for SI engine speed regulation. The proposed design exploits ALA’s adaptive energy-balance strategy to determine optimal controller gains that minimize a multi-objective cost function combining overshoot, setpoint error, and stability criteria. The study presents the first comprehensive integration of ALA into nonlinear 2-DOF PID tuning for engine speed control, positioning it as a novel contribution to both optimization and automotive control literature. A systematic review of prior works (summarized in Table 1 of this paper) reveals several unresolved challenges in the field of engine speed regulation:

1. Limited adaptability of classical and fractional-order PID controllers under nonlinear combustion and torque-load conditions.
2. Incomplete separation of setpoint tracking and disturbance rejection in most tuning frameworks, leading to performance trade-offs.
3. Lack of metaheuristic algorithms with adaptive search control capable of ensuring convergence stability across multiple independent runs.
4. Insufficient robustness testing, as many studies focus only on transient response and neglect trajectory-tracking and disturbance-rejection analyses.

To address these gaps, the present study proposes a comprehensive optimization-based control framework with the following core contributions:

- (i) Development of a nonlinear 2-DOF PID controller that enables independent tuning of setpoint-tracking and disturbance-rejection channels, improving adaptability under dynamic engine conditions.
- (ii) Integration of the ALA as a novel optimizer for controller parameter tuning, employing its energy-adaptive exploration–exploitation strategy to achieve consistent convergence across multiple independent runs.
- (iii) Formulation of a multi-term cost function that simultaneously minimizes percent overshoot, setpoint error, and a stability coefficient, ensuring fast yet smooth transient performance.
- (iv) Comprehensive evaluation through statistical robustness analysis (30 independent optimization trials), transient and steady-state time-response comparisons, trajectory-tracking tests under variable speed references, and disturbance-rejection simulations with sudden load torque perturbations.
- (v) Quantitative comparison against four recent metaheuristic algorithms—SFOA, PO, COA, and DMO—demonstrating the superior stability, precision, and reliability of the ALA-optimized controller.

**Table 1:** Summary of recent studies on PID controller optimization for engine speed regulation.

Category	References	Application Type	Controller Type	Optimization Algorithm	Key Contributions	Limitations
Engine and Automotive Applications	Ref^22^	Electric Vehicle (EV)	Linear & Nonlinear PID	AO	Improved tracking, robustness in EVs	Not tested on internal combustion engines
	Ref^2^	SI Engine	PI	BA	Superior performance in load/no-load	Basic controller type
	Ref^10^	Hybrid Vehicle Thermal System	2-DOF PID	PSO	Improved transient and steady-state	Focus on thermal management only
	Ref^21^	4-cylinder SI Engine	Filtered PID	AO	Outperformed DE, GWO, Simulink tuner	No trajectory or disturbance analysis
	Ref^1^	SI Engine	PID-F	EEFO	Lower overshoot, rise time, high robustness	Lacks 2-DOF flexibility
General Nonlinear Systems and Optimization Studies	Ref^15^	General Nonlinear Systems	2-DOF PID	GA	Independent tuning of setpoint/disturbance	Slower convergence vs. recent algorithms
	Ref^26^	General Optimization Problems	–	ALA	Superior convergence, accuracy, robustness	Not yet integrated with 2-DOF PID (this study addresses it)

The research methodology begins with a rigorous mathematical modeling of the SI engine speed control system, incorporating throttle flow dynamics, manifold pressure variation, combustion torque generation, and rotational motion equations. A detailed Simulink framework is developed to emulate realistic nonlinear behaviors including load torque, combustion delay, and throttle saturation effects. The ALA is implemented to tune the 2-DOF PID parameters within predefined bounds based on the proposed cost function. A sequence of analyses validates the proposed method’s performance. The statistical robustness examination confirms ALA’s consistent convergence and minimal standard deviation compared to SFOA, PO, COA, and DMO. The transient response evaluation highlights its superior rise and settling times with negligible overshoot. The steady-state response assessment shows near-zero steady-state error and high accuracy under constant reference conditions. The trajectory-tracking test demonstrates precise adaptability to varying reference speeds, replicating real driving scenarios. Finally, the disturbance-rejection analysis verifies exceptional resilience to external torque perturbations, confirming robust and energy-efficient control. By embedding the ALA within a nonlinear 2-DOF PID architecture, this work establishes a bio-inspired intelligent control framework that advances the state of the art in automotive speed regulation. The proposed controller achieves near-ideal performance metrics (rise time of 0.3114 s, settling time of 2.4313 s, overshoot of 0.0027%, and steady-state error of 2.62 × 10⁻¹¹%) surpassing all comparative algorithms under identical simulation conditions. These findings not only validate ALA’s robustness but also highlight its scalability for other nonlinear dynamic systems such as electric drives, renewable energy converters, and robotic actuators.

The remainder of the paper is organized as follows. Section 2 introduces the theoretical foundation and mathematical formulation of the artificial lemming algorithm. Section 3 presents the detailed modeling of the spark-ignition engine speed control system. Section 4 outlines the design of the nonlinear 2-DOF PID controller, the cost-function definition, and the simulation setup. Section 5 provides extensive comparative results, including statistical analyses, transient and steady-state performance, trajectory-tracking, and disturbance-rejection evaluations. Section 6 concludes the study by summarizing the main findings, discussing implementation perspectives, and proposing directions for future research in intelligent automotive control.

## Overview of artificial lemming algorithm

The artificial lemming algorithm (ALA) is a bio-inspired metaheuristic optimization algorithm that simulates four key behaviors of lemmings in nature. The ALA simulates essential lemming behaviors which include long-distance migration together with digging holes while searching for food and avoiding predators. The optimization process benefits from these behaviors as they strategically maintain balance between exploration and exploitation. The ALA integrates an energy-decreasing mechanism that adjusts exploration and exploitation phases dynamically to avoid premature convergence while enhancing global search capabilities. The ALA algorithm uses mathematical formulation to achieve efficient optimization in complex high-dimensional nonconvex environments. The following section demonstrates the math behind the four behaviors while explaining the transition mechanism that relies on energy considerations^26^.

## Mathematical formulation of ALA

### Initialization phase

ALA is a population-based algorithm where a set of candidate solutions are initialized within the problem’s search space. The initial population is represented as a matrix:

1 $$\:Z={\left[\begin{array}{ccc}{Z}_{11}&\:{Z}_{12}\dots\:&\:{Z}_{1Dim}\\\:{Z}_{\begin{array}{c}21\\\:.\\\:.\\\:.\\\:.\end{array}}&\:{Z}_{\begin{array}{c}22\\\:.\\\:.\\\:.\\\:.\end{array}}\dots\:&\:{Z}_{\begin{array}{c}2Dim\\\:.\\\:.\\\:.\\\:.\end{array}}\\\:{Z}_{N1}&\:{Z}_{N2}\dots\:&\:{Z}_{NDim}\end{array}\right]}_{N\times\:Dim}$$

Where $\:N$ is the population size, and Dim is the number of problem dimensions. Each decision variable $\:{z}_{i,j}$​ is initialized within predefined boundaries $\:\left[L{B}_{j},U{B}_{j}\right]\:$using:

2 $$\:{\varvec{z}}_{\varvec{i},\varvec{j}}=L{B}_{j}+rand\times\:\left(U{B}_{j}-L{B}_{j}\right),\:\:\:\:\:\:i=\text{1,2},3,...N\:\:\:\:and\:\:\:\:\:\:\:j=\text{1,2},3,\dots\:Dim$$

where rand is a random number in the range [0,1].

### Exploration behaviors phase

In this optimization method, long-distance migration and digging holes, allowing search agents to scan the search space and escape local optima guide exploration in ALA.

#### Long-distance migration

Lemmings undertake random migrations when resources are scarce. This behavior is modeled as:

3 $$\:{Z}_{i}\left(t+1\right)={Z}_{best}\left(t\right)+F\times\:BM\times\:\left(R\times\:\left({Z}_{best}\left(t\right)-{Z}_{i}\left(t\right)\right)+\left(1-R\right)\times\:\left({Z}_{i}\left(t\right)-{Z}_{a}\left(t\right)\right)\right)$$

Where, $\:{Z}_{best}$, $\:F$, $\:BM$, $\:R$ and $\:{Z}_{a}\left(t\right)$ are the best solution found so far, search direction flag, brownian motion, enhancing global search by introducing dynamic step sizes, random scaling vector and randomly selected search agent, respectivly. In addition, $\:F$ and $\:R$ is calculated as:

4 $$F = \left\{ {\begin{array}{*{20}{c}} {1,\:if\:\left\lfloor {2 \times \:rand + 1} \right\rfloor = 1} \\ {\:\:\:\:\:\: - 1,othewise} \end{array}} \right.$$

5 $$\:R=2\times\:rand\left(1,Dim\right)-1$$

#### Digging holes

Lemmings dig holes to create safe shelters, modeled as:

6 $$\:{Z}_{i}\left(t+1\right)={Z}_{i}\left(t\right)+F\times\:L\times\:\left({Z}_{best}\left(t\right)-{Z}_{b}\left(t\right)\right)$$

Where, $\:{Z}_{b}\left(t\right)$, $\:L$ are another randomly selected search agent and time-dependent digging coefficient. $\:L$ is defined as:

7 $$\:L=rand\times\:\left(1+sin\left(\frac{t}{2}\right)\right)$$

These two behaviors expand the search space coverage and help avoid local optima.

### Exploitation behaviors

Exploitation in ALA is achieved through foraging for food and evading predators, enabling local refinement around promising solutions.

#### Foraging for food

Lemmings search for food within a localized region. This is modeled using a spiral-wrapping search mechanism:

8 $$\:{Z}_{i}\left(t+1\right)={Z}_{best}\left(t\right)+F\times\:spiral\times\:rand\times\:{Z}_{i}\left(t\right)$$

Where, spiral and radius represent the foraging trajectory and the Euclidean distance between $\:{Z}_{i}\left(t\right)$ and$\:\:{Z}_{best}\left(t\right)$.

#### Evading predators

Lemmings evade predators by running back to their burrows. This is modeled as:

9 $$\:{Z}_{i}\left(t+1\right)={Z}_{best}\left(t\right)+F\times\:G\times\:Levy\left(Dim\right)\times\:\left({Z}_{best}\left(t\right)-{Z}_{i}\left(t\right)\right)$$

Where, $\:G$ and $\:Levy\left(x\right)$ are the escape coefficient and Lévy flight for random long jumps.$\:\:G$ and $\:Levy\left(x\right)$ are defined as:

10 $$\:G=2\times\:\left(1-\frac{t}{{T}_{max}}\right)$$

11 $$\:Levy\left(x\right)=0.01\times\:\frac{u\times\:\sigma\:}{{\left|v\right|}^{\frac{1}{\beta\:}}},\:\:\:\:\sigma\:={\left(\frac{{\Gamma\:}\left(1+\beta\:\right)\text{sin}\left(\frac{\pi\:\beta\:}{2}\right)}{{\Gamma\:}\frac{\left(1+\beta\:\right)}{2}\times\:{2}^{\frac{\left(\beta\:-1\right)}{2}}\times\:\beta\:}\right)}^{\frac{1}{\beta\:}}$$

Where, $\:\beta\:=1.5$, and $\:u$ and $\:v$ are ${\sim}U\left( {0,1} \right)$.

### Energy-Based balance

The transition between exploration and exploitation is controlled by an energy factor *E(t)*, which decreases over time:

12 $$\:E\left(t\right)=4\times\:arctan\left(1-\frac{t}{{T}_{max}}\right)\times\:\text{ln}\left(\frac{1}{rand}\right)$$

ALA switches between behaviors based on the threshold $\:E>1$:

13 $$\:\left\{\begin{array}{c}if\:E>1\:Exploration\:phase\\\:if\:E\le\:1\:\:Exploitation\:phase\end{array}\right.$$

### Computational complexity

The computational complexity of ALA is primarily determined by three main stages: initialization, fitness function evaluation, and the position updating of the lemmings. The complexity can be broken down as follows:

14 $$\:O\left(ALA\right)=O\left(N\right)+O\left({T}_{max}\times\:N\times\:Dim\right)+O\left({T}_{max}\times\:N\right)\approx\:O\left(N\times\:\left(1+{T}_{max}\times\:Dim+{T}_{max}\right)\right)$$

1. **Initialization**: The process of generating the initial population of $\:N\:$lemmings across $\:Dim$ dimensions has a complexity of $\:O\left(N\times\:Dim\right)$.
2. **Main Loop (Iterations)**: The core of the algorithm runs for a maximum of $\:{T}_{max}$ iterations. In each iteration:

- The fitness of each of the $\:N$ lemmings is evaluated, which contributes $\:O\left({T}_{max}\times\:N\right)$ to the complexity.
- The position of each of the $\:N$ lemmings is updated across all $\:Dim$ dimensions according to the algorithm’s behavioral rules (migration, digging, foraging, or evading). This is the most computationally intensive part, with a complexity of $\:O\left({T}_{max}\times\:N\times\:Dim\right)$.

Therefore, the total computational complexity of ALA is given by the sum of these components, which is dominated by the position update mechanism within the main loop. The overall complexity is approximately$\:O\left({T}_{max}\times\:N\times\:Dim\right)$. This linear relationship with respect to the population size $\:N$, the number of iterations $\:{T}_{max}$, and the problem’s dimensionality $\:Dim$ indicates strong scalability. It implies that the computational cost grows predictably and manageably as the problem size increases, avoiding the exponential growth seen in less scalable algorithms. This makes ALA a viable candidate for high-dimensional and complex optimization problems without incurring prohibitive computational overhead.

## Mathematical modeling of spark ignition (SI) engine speed control system

The spark ignition engine speed control system stands as a crucial component within internal combustion engine functionality. The SI engine generates power through a controlled spark event that ignites the air-fuel mixture to drive the crankshaft. Various factors including air intake flow, fuel combustion efficiency, and mechanical resistance contribute to controlling the engine speed. This section demonstrates the development of mathematical models which describe the speed dynamics in spark ignition engines.

### Model formulation

The model for the SI engine speed control system is composed of several key equations, each describing a specific aspect of the engine’s dynamics. Below, we detail each component, ensuring clarity and alignment with standard engineering practices.

#### Throttle flow dynamics

The mass flow rate of air through the throttle, denoted as $\:{m}_{ai}$, is modeled using the equation for isentropic flow through a restriction:

15 $$\:{\dot{m}}_{ai}=A\left(\alpha\:\right)\frac{2}{\vartheta\:-1}{P}_{a}{C}_{d}\frac{2}{\vartheta\:-1}\vartheta\:R{T}_{a}\left[{\left(\frac{{P}_{m}}{{P}_{a}}\right)}^{\frac{2}{\vartheta\:}}-{\left(\frac{{P}_{m}}{{P}_{a}}\right)}^{\frac{\vartheta\:+1}{\vartheta\:}}\right]$$

Where, $\:A\left(\alpha\:\right)$, $\:{P}_{a}$, $\:{C}_{d}$, $\:\vartheta\:$, $\:R$, $\:{T}_{a}$ and $\:{P}_{m}$ are effective throttle area as a function of throttle angle $\:\alpha\:\left({m}^{2}\right)$, ambient pressure, discharge coefficient, specific heat ratio of air, gas constant, ambient temperature and manifold pressure, respectivly. This equation quantifies air inflow based on throttle position, which is a primary control variable, while the pressure ratio dictates flow behavior. For subsonic flow $\:(\frac{{P}_{m}}{{P}_{a}}>0.528)$, this formulation holds. otherwise, choked flow conditions apply but are simplified here for common operating ranges.

#### System representation

An integrated network of components determines the speed dynamics of spark-ignition (SI) engines by working together to control engine performance. The throttle stands at the heart of this mechanism because it manages airflow into the intake manifold. The throttle position adjustment alters the volume of incoming air which affects engine power output through changes in combustion characteristics. The dynamics of intake manifold pressure determine the air-fuel mixture entering the combustion chamber which affects combustion efficiency and responsiveness.

During combustion chemical energy transforms into mechanical work which supplies the engine with the essential power needed for operation. The crankshaft transmits power by transforming the pistons’ reciprocating movement into a continuous rotational motion which powers the vehicle. The load torque reflects the external forces and resistance elements affecting the engine which include friction, drivetrain loads and auxiliary system demands. These forces affect engine speed stability and behavior which makes them essential for system dynamics operation. The dynamic relationship between these components shapes the performance and speed characteristics of the SI engine.

### Air intake and manifold pressure dynamics

The airflow rate into the intake manifold is a function of the throttle position$\:\:\alpha\:$, ambient pressure $\:{P}_{amb}$, and manifold pressure$\:{\:P}_{m}$:

16 $$\:{\dot{m}}_{ai}=f\left(\alpha\:,\:{P}_{amb},{\:P}_{m}\right)$$

Where, $\:{\dot{m}}_{ai}$, $\:\alpha\:$, $\:{P}_{amb}$ and$\:{\:P}_{m}$ are the mass flow rate of air into the intake manifold, the throttle opening angle, the ambient air pressure and the manifold pressure respectively. The manifold pressure changes over time as:

17 $$\:{\dot{P}}_{m}=\frac{RT}{{V}_{m}}\left({\dot{m}}_{ai}-{\dot{m}}_{ao}\right)$$

Where, $\:R$, $\:T$, $\:{V}_{m}$ and $\:{\dot{m}}_{ao}$ are the specific gas constant, the temperature of the air, the volume of the intake manifold and the mass flow rate of air leaving the manifold into the cylinders respectively.

### Combustion and torque generation

The engine torque $\:{T}_{eng}\:$is primarily influenced by the air-fuel mixture and the spark ignition event. The torque generated by the combustion process can be expressed as:

18 $$\:{T}_{eng}=f\left({m}_{a},\:AFR,\sigma\:,N\right)$$

Where, $\:{m}_{a},\:AFR,\sigma\:\:and\:N$ are the mass of air entering the cylinder, the air-fuel ratio, which defines combustion efficiency, the spark timing, crucial for optimal power output and engine speed respectively.

The combustion efficiency is influenced by the flame propagation rate, which depends on engine load and fuel characteristics. A delay in spark timing can reduce torque output and affect engine speed stability.

### Engine speed dynamics

The rotational motion of the crankshaft follows Newton’s second law:

19 $$\:J\dot{N}={T}_{eng}-{T}_{load}$$

Where, $\:J$, $\:N$ and $\:{T}_{load}$ are the moment of inertia of the rotating components, the engine acceleration and the external torque disturbances, such as friction and road load respectively.

### Analysis of SI engine speed behavior

Air intake regulation alongside combustion efficiency and external resistances govern the speed of spark-ignition (SI) engines through their complex interactions. The throttle response forms the central element of this dynamic system because changes in throttle position affect both airflow and manifold pressure which then modifies the torque output. The engine performance is modified through combustion efficiency changes which result from either advancing or retarding the spark ignition timing. Fluctuations in the engine’s speed occur due to external resistances which demand real-time adjustments to maintain stability as the engine responds to load torque variations. A thorough understanding of the mathematical relationships in SI engine speed control leads to improvements in fuel efficiency optimization and emission reduction while also strengthening system stability.

## Innovative control paradigm

### Fundamental of nonlinear 2-DOF PID controller

**Fig. 1:**
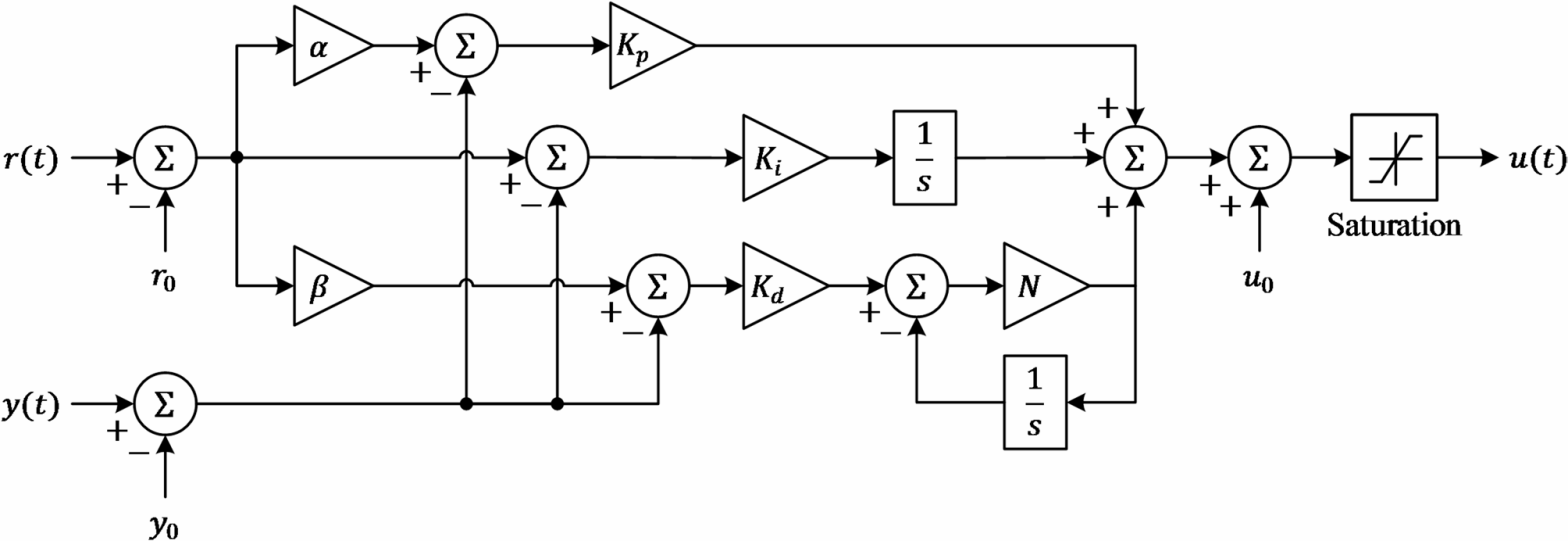
Block diagram of proposed 2-DOF PID controller with saturation for nonlinear system.

The two-degree-of-freedom (2-DOF) PID controller is an advanced control strategy designed to improve engine speed regulation in spark ignition (SI) engines by offering independent tuning of setpoint tracking and disturbance rejection. Unlike traditional PID controllers, which struggle to balance these objectives, the 2-DOF PID controller introduces separate gain factors to enhance adaptability and performance in nonlinear and dynamic environments. The inclusion of saturation handling ensures the controller remains stable despite physical constraints, making it well-suited for automotive applications where engine load variations, fuel injection dynamics, and external disturbances significantly impact speed regulation. 2-DOF PID control strategy optimized using the ALA to dynamically tune control parameters for optimal engine performance. The block diagram of the proposed controller is illustrated in Fig. 1, showcasing the key functional components.

The block diagram in Fig. 1 represents the proposed 2-DOF PID controller with saturation for nonlinear engine speed regulation. The controller is designed to precisely adjust the throttle position or fuel injection rate to maintain the desired engine speed$\:\:\omega\:\:\left(t\right)$. The major components of the controller can be listed as (1) reference input processing, (2) error computation and control actions, and (3) control law and mathematical formulation.

The desired engine speed $\:r\left(t\right)$ is modified using a feedforward gain $\:\alpha\:\:$before passing through the proportional control loop. Another independent gain factor, $\:\beta\:$, adjusts the reference input, ensuring a separate tuning mechanism for setpoint tracking. The error signal is computed as $\:e\left(t\right)=r\left(t\right)-y\left(t\right)$ where $\:y\left(t\right)$ represents the measured engine speed. The controller includes PID components, each contributing to the control response:

- **Proportional term (**$\:{\varvec{K}}_{\varvec{p}}$**​)**: The proportional action is modified by $\:\alpha\:$ to control how much of the reference input $\:R\left(s\right)\:$contributes to the proportional error. This allows for better tracking of setpoints. The proportional term is given by $\:{K}_{p}\left(\alpha\:R\left(s\right)-Y\left(s\right)\right)$.
- **Integral term (**$\:{\varvec{K}}_{\varvec{i}}$**​)**: The integral action accumulates the error over time, ensuring zero steady-state error for step inputs. It acts on the difference $\:R\left(s\right)-Y\left(s\right).$.

20 $$\:{K}_{i}\frac{1}{s}\left(R\left(s\right)-Y\left(s\right)\right)$$

- **Derivative term (**$\:{\varvec{K}}_{\varvec{d}}$**​)**: The derivative action improves transient response and stability by considering the rate of change of error. In 2-DOF PID, the derivative action is applied to, allowing finer tuning of how the reference input affects the response.

21 $$\:{K}_{d}\frac{s}{1+\frac{s}{N}}\left(\beta\:R\left(s\right)-Y\left(s\right)\right)$$

The control signal $\:U\left(s\right)$ generated by the 2-DOF PID controller follows the equation:

22 $$\:U\left(s\right)={K}_{p}\left[\alpha\:\left(R\left(s\right)-Y\left(s\right)\right)\right]+{K}_{i}\frac{1}{s}\left(R\right(s)-Y(s\left)\right)+{K}_{d}\frac{s}{1+\frac{s}{N}}\left(\beta\:R\left(s\right)-Y\left(s\right)\right)$$

where, $\:U\left(s\right)$, $\:R\left(s\right)$, $\:Y\left(s\right)$, $\:N$, $\:{K}_{p}$, $\:{K}_{i}$, $\:{K}_{d}$, $\:\beta\:$ and $\:\alpha\:$ are the control output, the reference input, the process output, the derivative filter coefficient, the proportional, integral, derivative gains and weighting factors respectively.

### Design and simulation of an engine speed control system in simulink

**Fig. 2:**
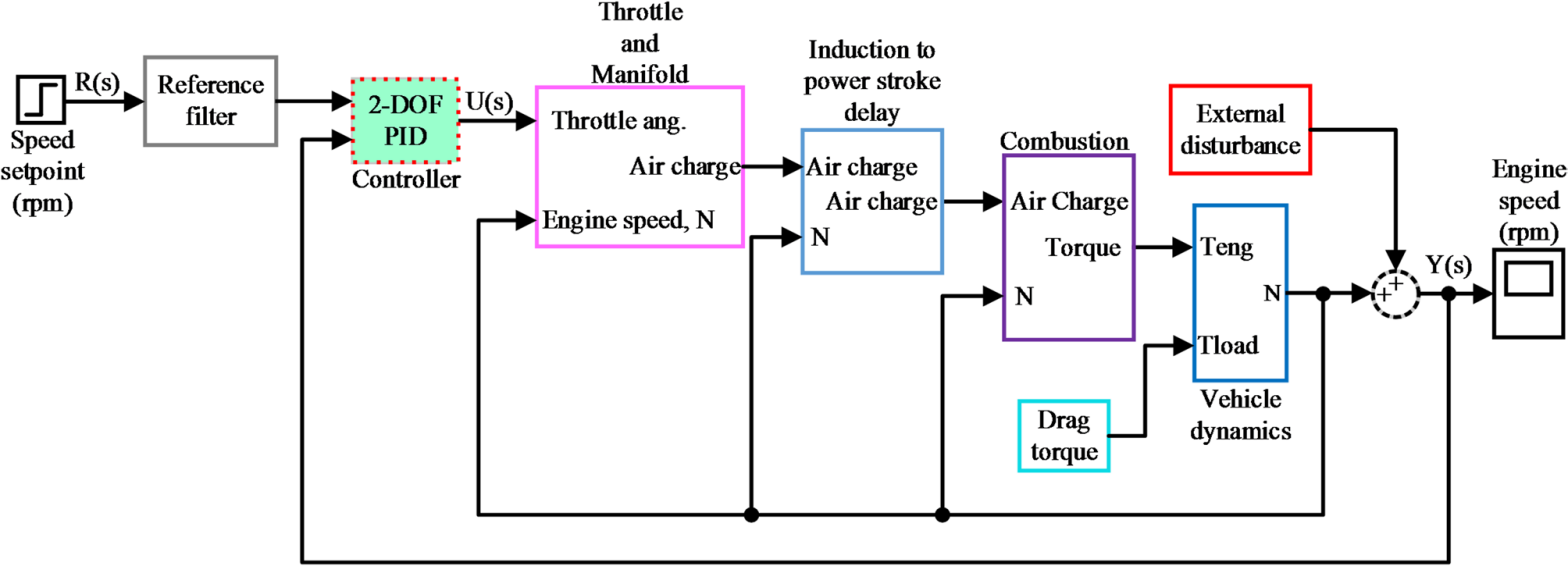
Simulink model of complete system.

The Simulink model depicted in Fig. 2 demonstrates a closed-loop control system for internal combustion engine speed regulation which combines multiple subsystems to achieve effective engine speed management by responding to disturbances and changing vehicle conditions. To maintain a desired speed setpoint input the system modifies the throttle angle using a 2-DOF PID controller for precise regulation. The reference filter modifies the input setpoint to ensure gradual transitions while preventing system destabilization from sudden changes. The control signal from the controller establishes the throttle angle which regulates the amount of air charge entering the engine manifold.

The throttle and manifold subsystem adjusts air intake through the control signal that regulates engine speed. The inherent delay in engine dynamics creates a time gap between air intake and combustion which requires the introduction of an induction-to-power stroke delay to model this delay. The delayed air charge proceeds to the combustion stage and transforms into engine torque there. The engine torque produced during combustion depends on the air-fuel ratio and engine speed because both variables change dynamically during system operation.

The engine torque resulting from combustion affects vehicle dynamics by combining with both load torque and drag torque. External resistances include road friction alongside air drag and the vehicle’s own inertia. The external disturbance block manages additional disturbances such as road slope variations and sudden accelerations by producing unpredictable effects that require compensation from the controller. Real-time measurement of engine speed provides feedback to the controller so it can close the loop and adjust dynamically for stable performance.

The system functions via a continuous feedback mechanism where the controller dynamically adapts throttle position as engine speed changes along with external disturbances and load variations. The engine maintains a consistent speed despite driving conditions that fluctuate. The combination of delay model integration with combustion management and vehicle dynamics alongside disturbance handling establishes a robust engine control system which effectively manages engine speed across diverse load conditions and external factors.

### Optimization problem

A defined cost function serves as the foundation for formulating the optimization problem for controller tuning because it evaluates control system performance. Key performance metrics within the cost function include normalized percent overshoot, a stability coefficient, and the output-setpoint error. The effectiveness of the controller to deliver optimal performance while ensuring system stability is evaluated through these metrics. Therefore, the following cost function was chosen for this study:

23 $$\:{F}_{cost}=\left(1-{\uprho\:}\right)\times\:{PO}_{norm}+\rho\:\times\:\underset{0}{\overset{{t}_{f}}{\int\:}}\left|e\right(t\left)\right|dt$$

where, $\:{F}_{cost}$, $\:{PO}_{norm}$, $\:e\left(t\right)$, $\:\rho\:$ and $\:{t}_{f}$ are the overall cost function value, the normalized percent overshoot, error, weighting coefficient that determines the trade-off between overshoot minimization and error reduction and the final simulation time, respectively. In addition, simulation time$\:\:{t}_{f}=50s\:$, stability coefficient$\:\:\rho\:=\frac{1}{50}$, and $\:e\left(t\right)\:$is the error between the setpoint and output. The simulation time determines the duration for which the optimization process operates to verify the performance criteria of the controller under the specified constraints. The goal is to reduce the cost function by altering the controller parameters inside their preset tuning limits detailed in Table 2.

**Table 2:** Limits of 2-DOF PID controller.

Parameter	Tuning range
$\:{\varvec{K}}_{\varvec{p}}$	$\:[0.001-0.25]$
$\:{\varvec{K}}_{\varvec{i}}$	$\:[0.001-0.1]$
$\:{\varvec{K}}_{\varvec{d}}$	$\:[0.001-0.01]$
$\:\varvec{N}$	$\:[100-2000]$
$\:\varvec{\alpha\:}$	$\:[0.8-1.2]$
$\:\varvec{\beta\:}$	$\:[0.1-10]$

**Fig. 3:**
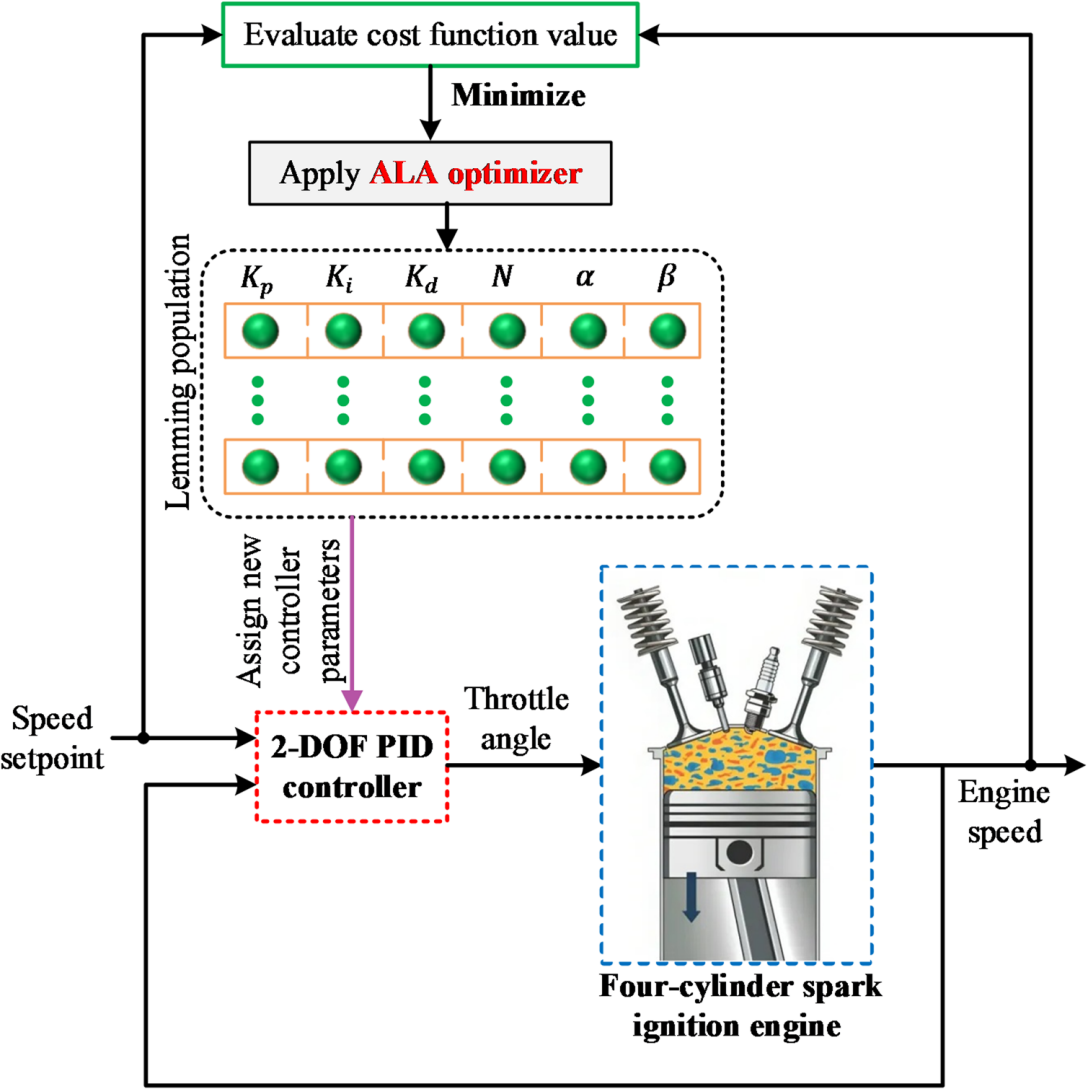
Block diagram of ALA-based engine speed control system.

The proposed cost function focuses on the primary dynamic-performance indices, overshoot, output–setpoint error, and stability to achieve precise and rapid speed regulation. An explicit weighting term for control effort (for instance, actuator energy) was intentionally omitted. In the considered spark-ignition engine, throttle actuation operates within limited angular ranges and low power demand, making energy consumption negligible relative to dynamic accuracy objectives. Moreover, excessive penalization of control effort could restrict throttle movement, slowing transient response and counteracting the goal of fast regulation. To ensure physical feasibility, actuator saturation limits (0°–90°) were already enforced, which inherently bounds control effort. Hence, omitting a separate control-effort term preserves responsiveness without compromising realistic actuator behavior. The tuning process utilizes an ALA that explores the parameter space systematically to find the best possible values. The ALA-based engine speed control system is depicted in Fig. 3, illustrating the interaction between the controller and the engine dynamics. This block diagram provides a visual representation of the system architecture, highlighting the flow of signals between different components, such as the controller, plant model, and optimization algorithm.

## Statistical analysis and nonlinear time-domain simulation results

### Statistical robustness examination of ALA optimizer

To evaluate the performance and robustness of the ALA optimizer, a comparative analysis was conducted against recent metaheuristic algorithms, including the starfish optimization algorithm (SFOA)^24^, parrot optimizer (PO)^18^, coati optimization algorithm (COA)^16^, and dwarf mongoose optimization algorithm (DMO)^23^. The statistical evaluation was performed over 30 independent runs, ensuring a reliable assessment of optimization performance. Each run was executed with a predefined maximum number of iterations$\:{\:T}_{max}=50$ and a fixed population size$\:\:N=25$. Each of the 30 independent runs was initialized with a unique random seed, resulting in distinct initial populations while keeping the population size and algorithmic parameters fixed. This procedure ensured that the statistical evaluation reflects true stochastic variability rather than deterministic repetition.

#### Statistical boxplot analysis

**Fig. 4:**
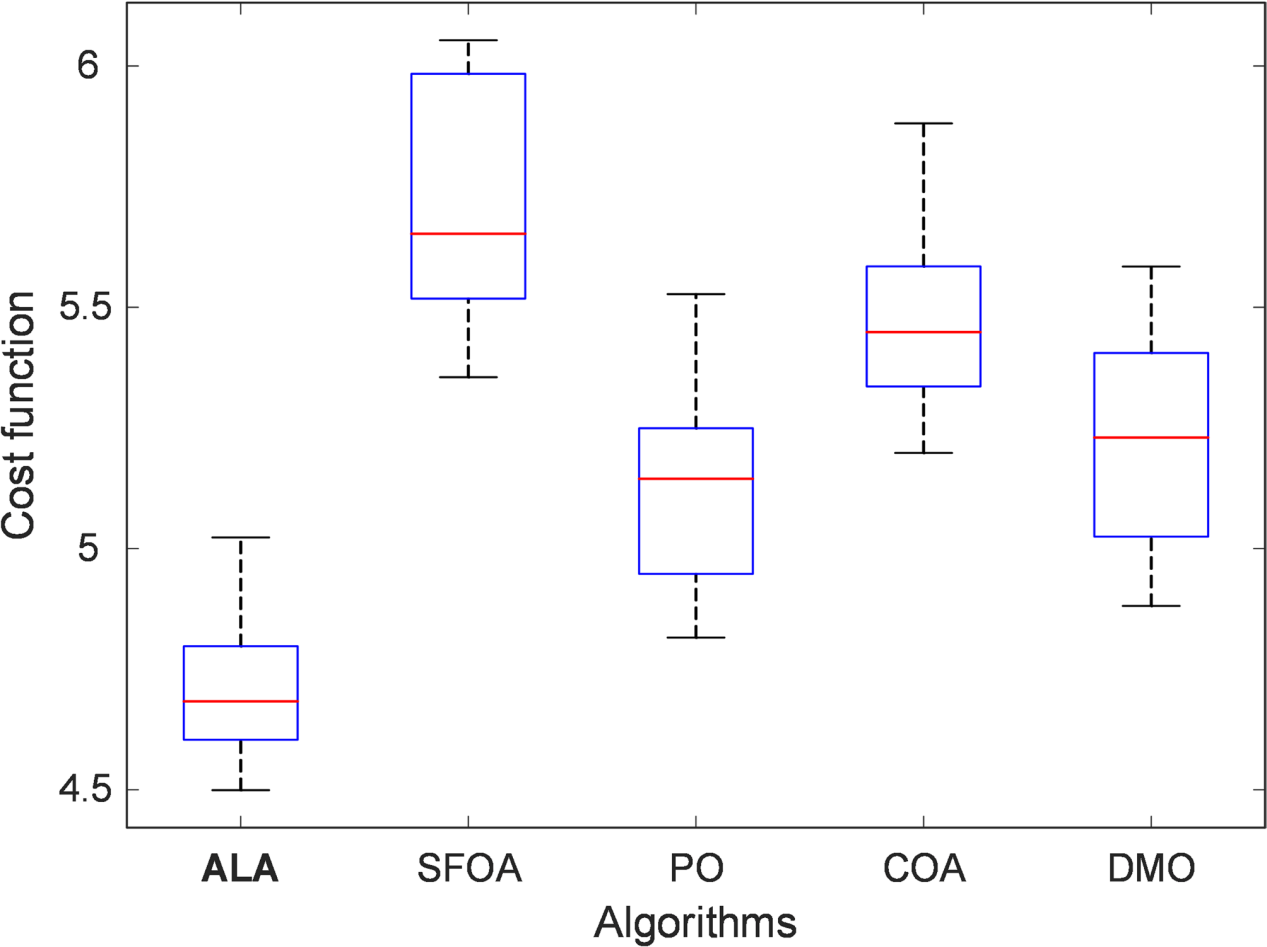
Statistical boxplot analysis of ALA, SFOA, PO, COA and DMO.

Figure 4 presents the boxplot distribution of the cost-function outcomes obtained by all competing algorithms. This graphical representation highlights both the central tendency and the dispersion of the results across the 30 trials. The ALA demonstrates the most compact distribution, characterized by a narrow interquartile range and closely spaced whiskers, signifying a stable and repeatable convergence toward near-optimal solutions. The median cost value of ALA lies well below those of the other algorithms, underscoring its superior mean performance. In contrast, SFOA and COA exhibit visibly larger interquartile spreads and several upper-range outliers, indicating inconsistent convergence and a higher likelihood of premature stagnation in local optima. The PO algorithm performs moderately, showing a narrower range than SFOA but still wider than ALA. The DMO also displays notable variability, implying weaker control of convergence dynamics and sensitivity to random initialization. These comparative trends confirm that ALA maintains both statistical stability and robustness, avoiding erratic behavior that commonly affects other metaheuristics. The observed consistency in its boxplot distribution reflects the effectiveness of ALA’s adaptive energy-driven mechanism, which dynamically regulates the transition between exploratory and exploitative phases.

#### Key statistical measures

The quantitative metrics summarized in Table 3 reinforce the visual findings of the boxplot. Among all algorithms tested, ALA achieved the lowest mean cost-function value (4.7170) and the smallest standard deviation (0.1429), evidencing its high convergence precision and robustness. The best result obtained by ALA (4.4993) is substantially lower than those of its competitors, while even its worst case (5.0230) remains superior to the best performances of several alternative methods. These results position ALA as the top-ranked optimizer (Rank 1), followed by PO (Rank 2), DMO (Rank 3), COA (Rank 4), and SFOA (Rank 5). The narrow gap between the best and worst ALA outcomes (approximately 0.52) demonstrates that the algorithm consistently converged to a similar fitness region across all independent runs. This robustness can be attributed to the energy-based balancing strategy integrated into ALA’s search process. By progressively reducing the energy coefficient during iterations, the algorithm transitions smoothly from broad global exploration to fine local exploitation, thereby minimizing random divergence and reducing the risk of entrapment in suboptimal regions. In comparison, the wider standard deviations of SFOA (0.2345), PO (0.1992), COA (0.1966), and DMO (0.2139) reveal higher stochastic variability and less predictable convergence. These algorithms occasionally yield promising results but fail to sustain consistency over repeated trials, often due to over-exploration or inadequate parameter adaptation. Taken together, both the statistical visualization and numerical analysis confirm that the ALA optimizer exhibits the highest degree of robustness and reliability among the evaluated algorithms. Its stable convergence behavior and superior cost-function performance provide a solid foundation for effectively tuning the nonlinear 2-DOF PID controller parameters in the subsequent control-system design stages.

**Table 3:** Evaluation of key statistical measures.

Measure	ALA	SFOA	PO	COA	DMO
Mean	4.7170	5.7088	5.1241	5.4855	5.2205
Standard deviation	0.1429	0.2345	0.1992	0.1966	0.2139
Best	4.4993	5.3551	4.8155	5.1981	4.8811
Worst	5.0230	6.0534	5.5272	5.8811	5.5845
Rank	1	5	2	4	3

### Cost function evolution curve

**Fig. 5:**
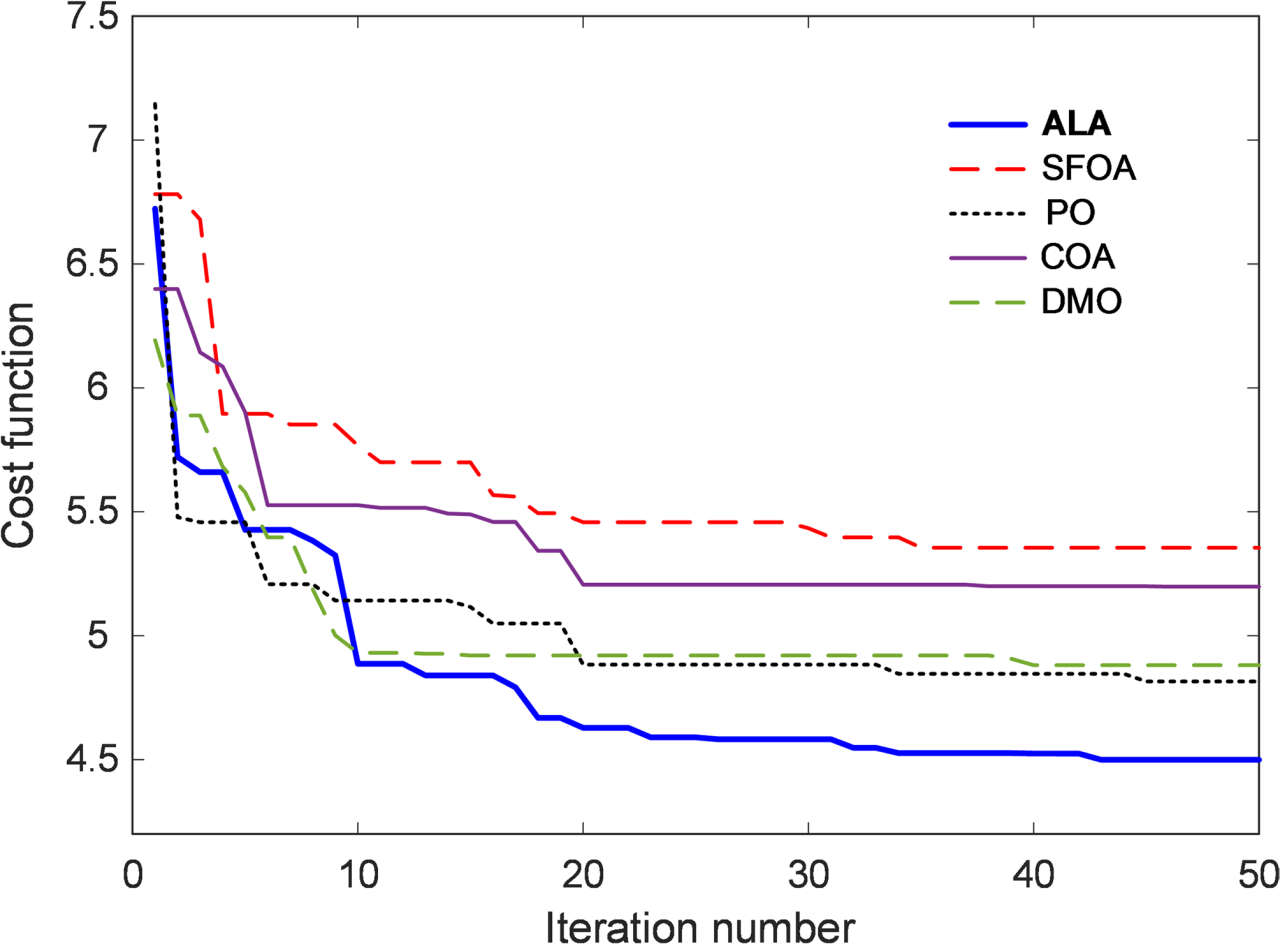
Change of $\:{F}_{cost}$ cost function.

The evaluation of the ALA optimizer along with other metaheuristic algorithms required an analysis of how the cost function changed during each iteration. The cost function serves as an essential metric for evaluating optimization effectiveness while offering information about how quickly solutions converge and their quality. The cost function evolution curve displayed in Fig. 5 demonstrates how quickly each algorithm reaches an optimal solution. The comparative analysis demonstrates that tested algorithms show distinct patterns of convergence behavior. Although the present cost-function evolution curves in Fig. 5 were generated using a fixed population size ($\:N=25$), preliminary trials and existing ALA studies suggest that convergence speed improves slightly with larger populations owing to enhanced search diversity, at the expense of higher computational cost. In contrast, very small populations may slow convergence or increase the risk of premature stagnation. Hence, $\:N=25$ offers a balanced trade-off between convergence speed and computational efficiency in this work.

#### The best 2-DOF PID controller parameters

The optimal parameters obtained for the 2-DOF PID controller using different optimization algorithms are presented in Table 4. These parameters include key control gains and system characteristics that contribute to the overall control performance. The results indicate that the ALA optimizer consistently outperforms other algorithms, achieving a lower cost function value, higher fitness score, and relatively low computational time. The cost function evolution curve further reinforces the robustness of ALA in optimizing 2-DOF PID controller parameters efficiently.

**Table 4:** Best 2-DOF PID controller parameters.

Parameter	ALA	SFOA	PO	COA	DMO
$\:{\varvec{K}}_{\varvec{p}}$	0.15030	0.10937	0.14073	0.18501	0.10570
$\:{\varvec{K}}_{\varvec{i}}$	0.056806	0.072681	0.052470	0.060403	0.056006
$\:{\varvec{K}}_{\varvec{d}}$	0.0072887	0.0096490	0.0081798	0.0092425	0.0060728
$\:\varvec{N}$	480.38	1422.2	1378.3	1604.8	624.97
$\:\varvec{\alpha\:}$	0.97255	0.92416	0.97101	0.96543	0.96225
$\:\varvec{\beta\:}$	1.8160	2.8316	2.0806	1.2038	2.5437

### Time response of closed-loop engine speed control system

The performance of the closed-loop engine speed control system was evaluated through time-domain response analysis. The transient and steady-state behavior of the system were examined to assess its stability and efficiency.

#### Transient response analysis

**Fig. 6:**
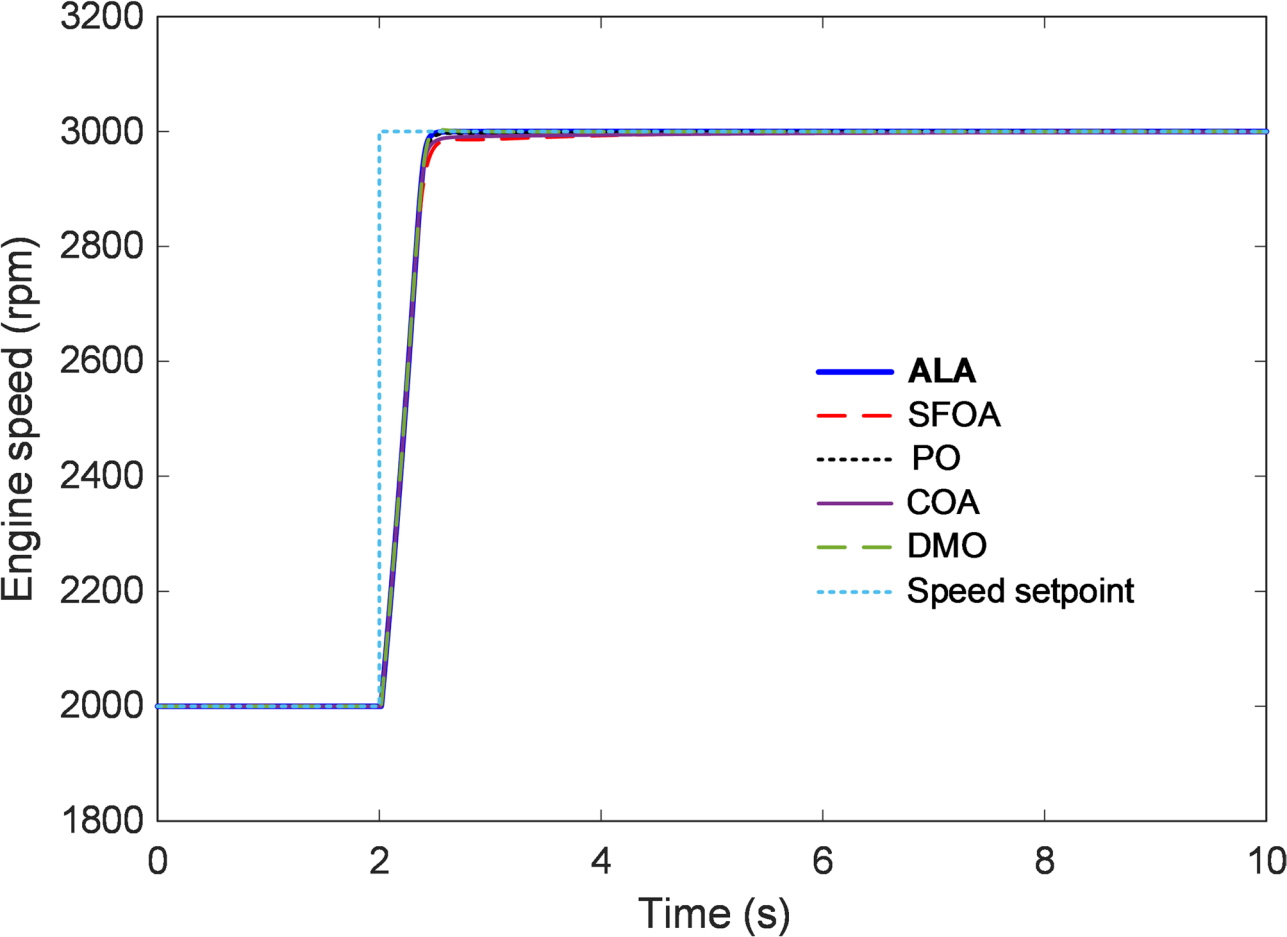
Transient response analysis.

**Fig. 7:**
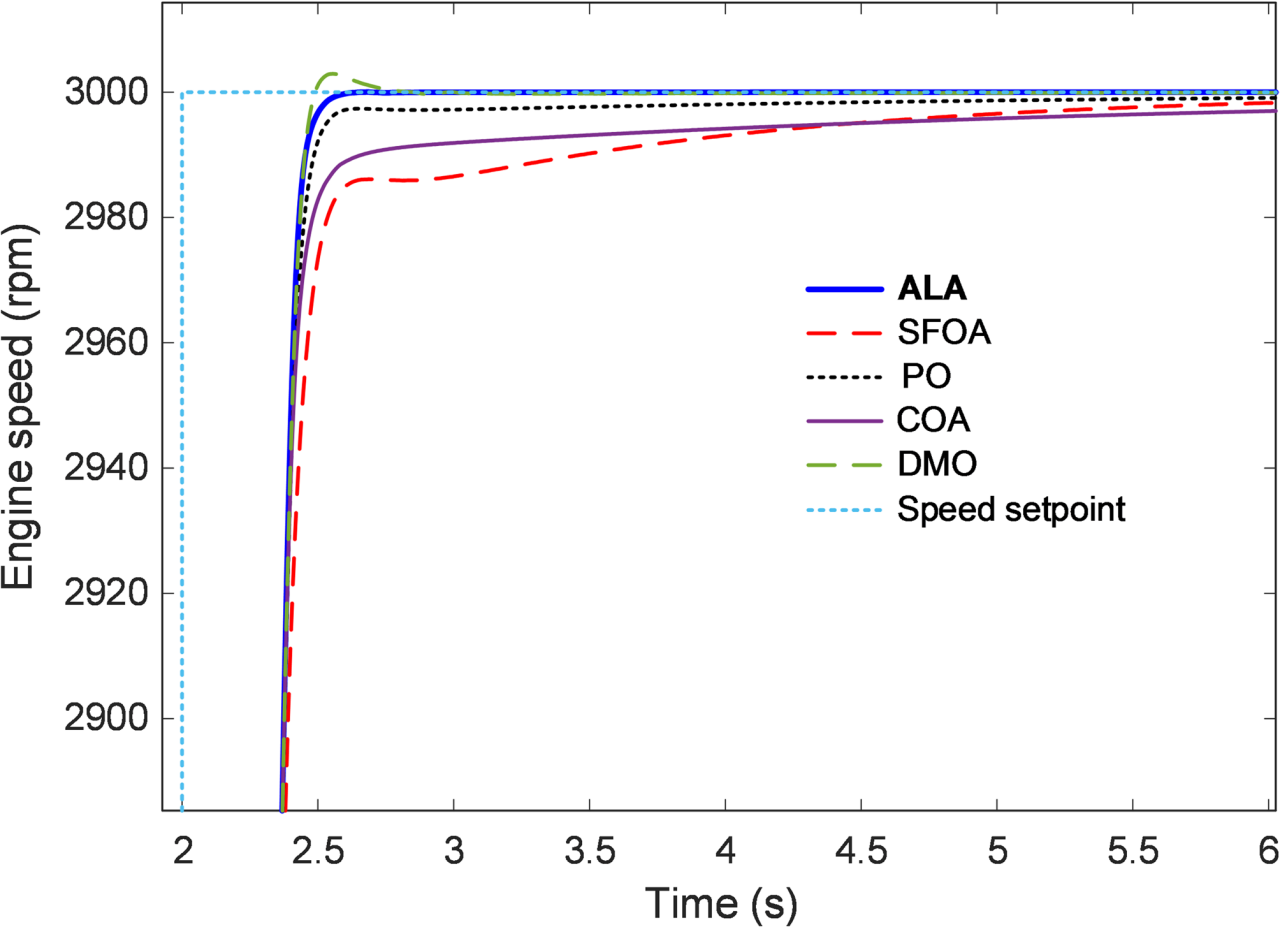
Enlarge view of Fig. 6.

The transient response analysis was conducted to evaluate how efficiently each optimized controller guides the engine speed toward the desired setpoint following a step-input change. Figures 6 and 7 illustrate the dynamic responses of the proposed ALA-optimized nonlinear 2-DOF PID controller compared with the SFOA, PO, COA, and DMO-based designs. The responses provide clear insight into the rise time, overshoot, and settling behavior that characterize the control system’s ability to achieve rapid and stable speed regulation under nonlinear conditions. As shown in Fig. 6, all controllers exhibit stable convergence toward the 3000 rpm reference following the step change at approximately t = 2 s. However, noticeable differences arise in their transient behaviors. The ALA-tuned controller demonstrates the most rapid rise and smoothest transition, attaining the target speed with virtually no overshoot or oscillation. This indicates that the ALA effectively balanced the proportional, integral, and derivative components of the 2-DOF PID structure, enabling fast energy transfer within the throttle–manifold–torque loop without inducing excessive control effort. In contrast, the SFOA-based controller exhibits a slower rise with visible lag, taking longer to settle within the steady-state band. The PO and COA controllers show comparable but slightly inferior responses, characterized by minor overshoots and small residual oscillations before stabilization. The DMO presents the weakest transient performance, marked by a slower approach and a pronounced undershoot during the acceleration phase, suggesting limited adaptability in managing the nonlinearities of the combustion dynamics.

To better visualize these distinctions, Fig. 7 magnifies the transient phase between 2 s and 6 s. The enlarged view highlights the superior smoothness of the ALA trajectory, which reaches the setpoint almost monotonically, confirming its effective damping of oscillatory components. The SFOA and COA controllers exhibit small amplitude ripples immediately after the rise, while the PO and DMO responses settle at slower rates with observable deviations from the reference.

#### Steady-state response analysis

**Fig. 8:**
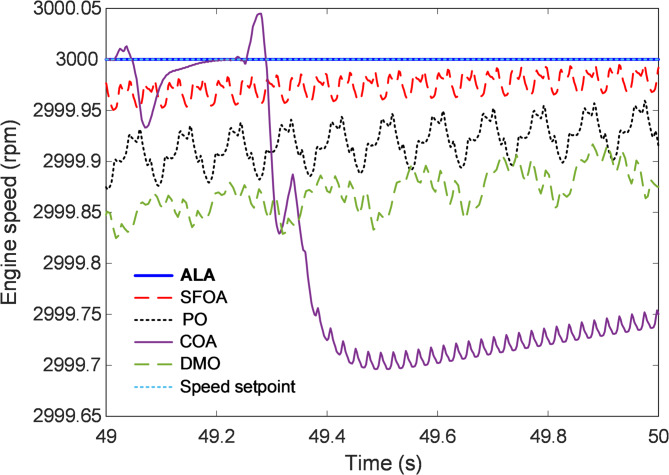
Steady-state response analysis.

The steady-state behavior of the closed-loop engine speed control system provides crucial insight into the long-term accuracy and stability of the designed controllers once the transient effects subside. Figure 8 illustrates the steady-state response of the proposed ALA-optimized nonlinear 2-DOF PID controller compared with those tuned by the SFOA, PO, COA, and DMO algorithms, under a fixed engine speed reference of 3000 rpm. The analysis focuses on the system’s ability to maintain the desired speed with minimal oscillation, negligible steady-state error, and high robustness against small residual disturbances. As depicted in the figure, the ALA-based controller maintains an exceptionally stable response that tightly follows the speed setpoint. Once the transient effects dissipate, the ALA curve exhibits a nearly flat trajectory with no visible deviation from the reference, confirming its superior steady-state accuracy. In contrast, the SFOA-based controller presents small but persistent oscillations around the setpoint, indicating incomplete attenuation of low-frequency fluctuations. The PO and COA responses show more pronounced ripples and a lower equilibrium speed, which reflect limited precision in regulating minor variations in engine torque. The DMO algorithm displays the poorest steady-state performance, with a significant undershoot followed by slow recovery, suggesting inadequate disturbance rejection capability and insufficient control gain adaptation.

#### Numerical evaluation of stability metrics

To quantitatively compare the performance of different optimization algorithms, Table 5 presents the numerical values of key normalized stability metrics, including rise time, settling time, overshoot, and steady-state error. In addition, rise time is measured from 10% to 90% of the final value, settling time is calculated within a ± 2% tolerance band, and steady-state error is evaluated at the end of $\:{t}_{f}$. The ALA optimizer achieved the shortest rise time (0.31139 s), indicating a faster initial response compared to other algorithms. It also demonstrated superior settling time (2.4313 s), ensuring rapid stabilization. ALA exhibited minimal overshoot (0.0027357%), significantly outperforming competing algorithms, which had notably higher overshoot percentages. Additionally, the ALA optimizer nearly eliminated steady-state error (2.6235E − 11%), showcasing high precision and control accuracy. These results indicate that the ALA optimizer provides the most stable and efficient engine speed control among the tested methods, achieving minimal transient deviations and superior steady-state accuracy. It is worth noting that the throttle actuator was modeled with physical saturation limits between 0° and 90°. Throughout all transient tests, the throttle position remained within these limits, reaching a maximum of 84.6% opening during load disturbances. This confirms that the controller operates within realistic actuator constraints, ensuring feasible and physically consistent performance.

**Table 5:** Numerical values of normalized stability metrics for ALA, SFOA, PO, COA and DMO.

Stability metric	ALA	SFOA	PO	COA	DMO
Normalized rise time (s)	0.31139	0.32763	0.31435	0.31329	0.31362
Normalized settling time (s)	2.4313	2.5354	2.4505	2.4809	2.4344
Normalized overshoot (%)	0.0027357	0.12677	0.047613	0.068074	0.29266
Normalized steady-state error (%)	2.6235E − 11	0.00080788	0.0085083	0.024995	0.012487

### Examination of controller output’s dynamic response

**Fig. 9:**
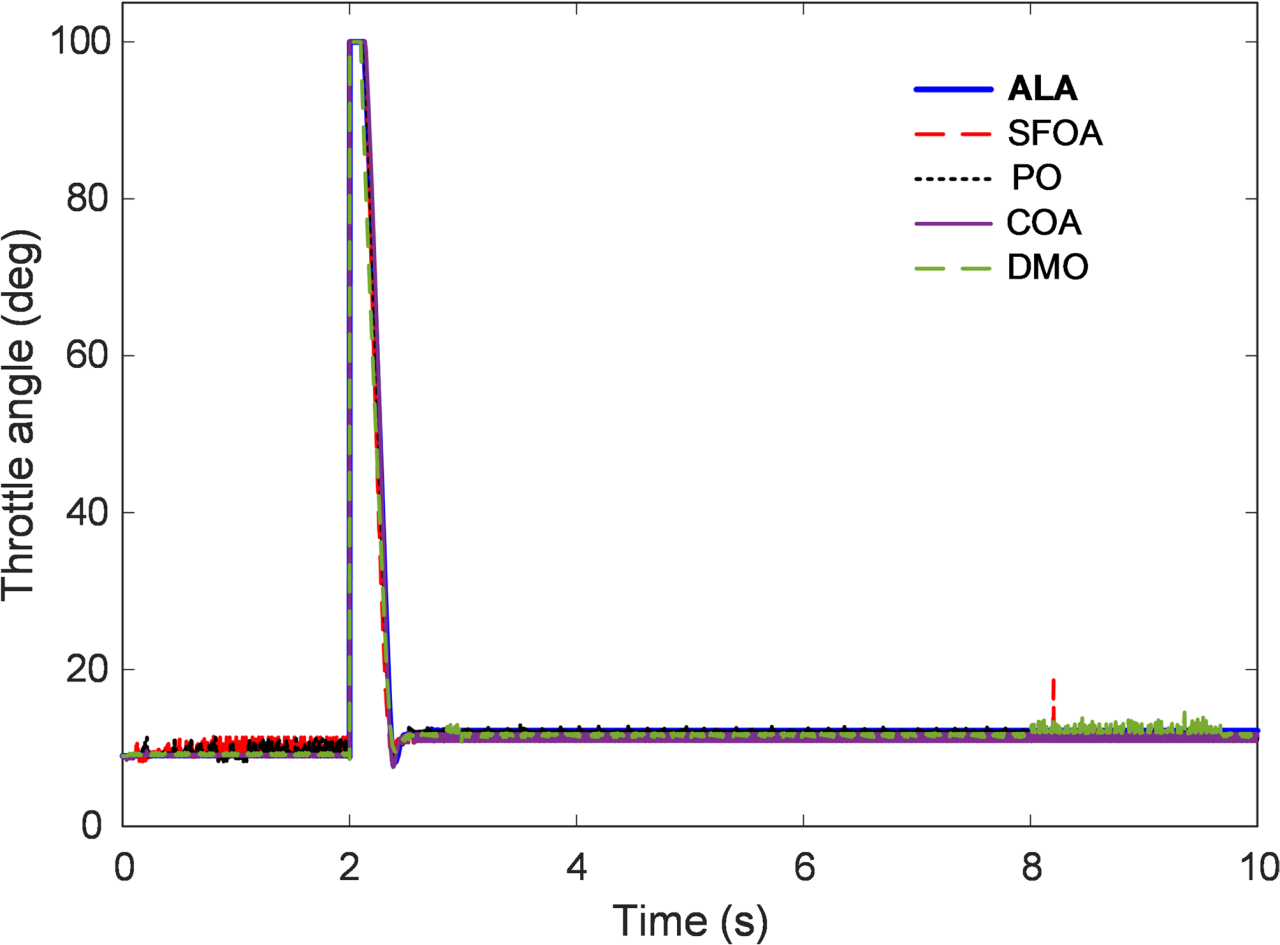
Time response of controller output.

**Fig. 10:**
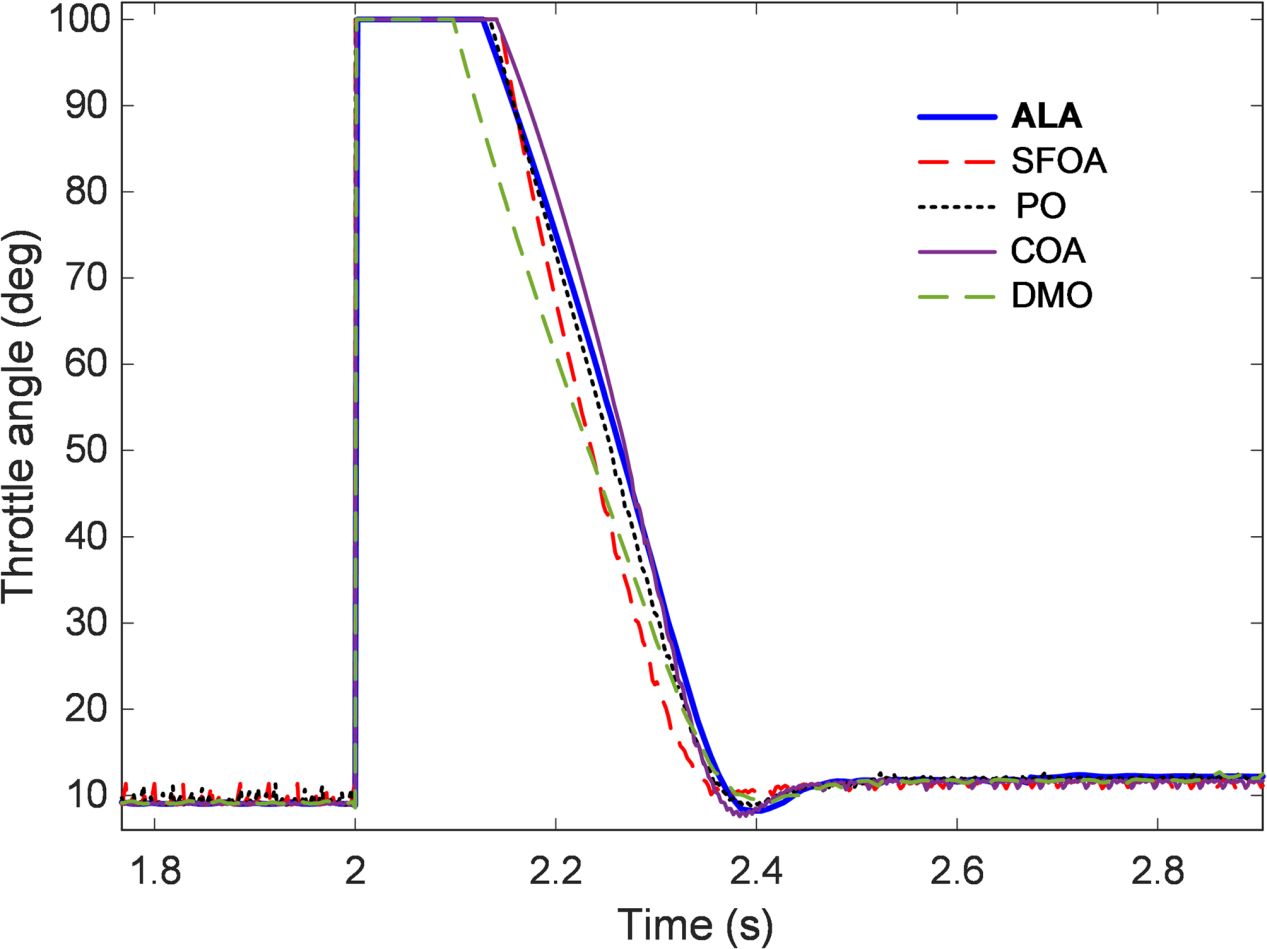
Enlarged view of Fig. 9.

The dynamic behavior of the controller output provides an essential indication of how effectively the control system manipulates the throttle actuator to maintain stable engine operation. Since throttle angle directly governs the airflow entering the combustion chamber, its temporal evolution reveals the controller’s capability to respond rapidly to speed demands while ensuring smooth actuator transitions. Figures 9 and 10 illustrate the throttle-angle variation over time for controllers tuned using ALA, SFOA, PO, COA, and DMO algorithms, allowing comparative evaluation of control signal smoothness, stability, and physical feasibility. As depicted in Fig. 9, all controllers respond promptly to the step change in the speed reference applied at approximately $\:t=2\:s$, with a sharp increase in throttle opening followed by a rapid return to steady-state operation. This pattern corresponds to the air-intake adjustment required to accelerate the engine from the lower speed region to the target of 3000 rpm. The ALA-optimized controller exhibits the most controlled and well-damped response, smoothly commanding the throttle to its peak angle near 100° before quickly stabilizing around the steady-state operating range. The absence of oscillations or overshoot in the ALA curve demonstrates that the actuator effort was both sufficient and efficiently regulated, avoiding unnecessary throttle fluctuations that could induce instability or increased fuel consumption. In comparison, the SFOA and PO controllers exhibit slightly less smooth responses, with small-amplitude fluctuations following the primary actuation phase. These oscillations indicate minor control energy inconsistencies and less effective damping. The COA response remains moderately stable but shows delayed convergence toward the steady-state angle. Conversely, the DMO-based controller presents the least desirable performance, characterized by small but persistent variations in throttle position throughout the steady phase, reflecting limited tuning precision and a weaker suppression of nonlinear air–fuel coupling effects.

To provide further clarity, Fig. 10 presents a magnified view of the throttle response between $\:t=1.8$ s and $\:t=2.8\:s$, capturing the transient phase in detail. The close-up comparison reveals that the ALA controller commands a sharp yet precisely regulated throttle closure immediately after the acceleration phase, maintaining the shortest stabilization interval among all tested methods. The SFOA and COA controllers exhibit slower recovery with slightly more oscillatory deceleration paths, whereas the PO and DMO signals display lingering ripples that may contribute to micro-fluctuations in manifold pressure. Quantitatively, the ALA-based controller achieves rapid actuation with negligible control overshoot and operates consistently within the predefined throttle limits (0°–90°). The effective energy distribution and adaptive search behavior of the ALA during optimization allowed it to fine-tune the derivative and integral gains of the 2-DOF PID structure, minimizing throttle chatter while sustaining fast responsiveness. This balance between agility and stability demonstrates the controller’s ability to handle the nonlinearities of the throttle and manifold subsystems without compromising mechanical or control smoothness.

### Trajectory tracking analysis

**Fig. 11:**
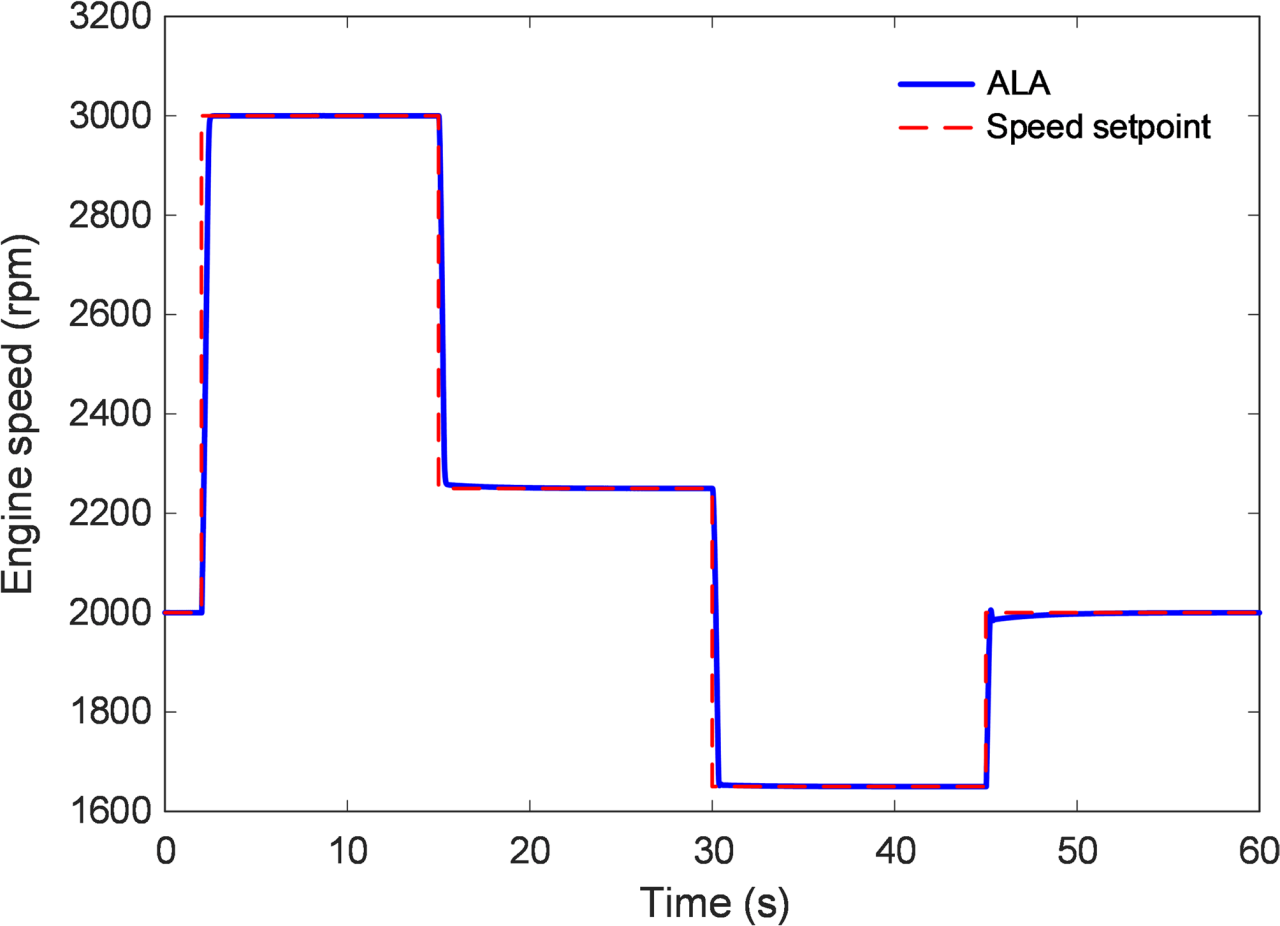
Trajectory tracking of proposed ALA-based 2-DOF PID controlled system under variable speed setpoint.

Trajectory-tracking evaluation was performed to assess the capability of the proposed ALA-based nonlinear 2-DOF PID controller to follow time-varying engine-speed references with high accuracy and minimal deviation. Unlike a single step-response test, this scenario subjects the controller to a sequence of abrupt speed-setpoint variations, thereby examining its adaptability and robustness under dynamic driving conditions. As illustrated in Fig. 11, the engine-speed reference was varied in several stages from approximately 2000 rpm to 3000 rpm, then decreased to 2250 rpm and 1650 rpm, followed by a recovery to around 2000 rpm. Throughout these transitions, the ALA-optimized controller demonstrated an almost perfect correspondence with the reference trajectory, exhibiting negligible lag and overshoot across all operating segments. The smooth transitions between successive speed levels indicate that the controller effectively balanced responsiveness with damping, ensuring continuity in torque generation and preventing abrupt throttle or manifold pressure changes. The minimal deviation observed between the actual speed and the commanded setpoint confirms that the ALA-based 2-DOF PID structure achieved both fast adaptation and excellent steady-state precision. Its dual-loop configuration enabled independent tuning of setpoint-tracking and disturbance-rejection channels, allowing rapid compensation for nonlinearities and parameter variations inherent in the combustion and load-torque dynamics. Consequently, the controller maintained close alignment with the desired trajectory even during sharp acceleration and deceleration phases, highlighting its robustness to system nonlinearities. Quantitatively, the average tracking error remained nearly zero over the entire 60 s simulation interval, while transient deviations during setpoint shifts were rapidly attenuated. No observable overshoot or oscillatory artifacts were present, confirming that the control effort was well-regulated within physical throttle limits. The adaptive exploration–exploitation mechanism of the ALA ensured optimal gain allocation across different operating regions, producing smooth actuator behavior without performance degradation at low or high speed ranges.

### Disturbance rejection analysis

**Fig. 12:**
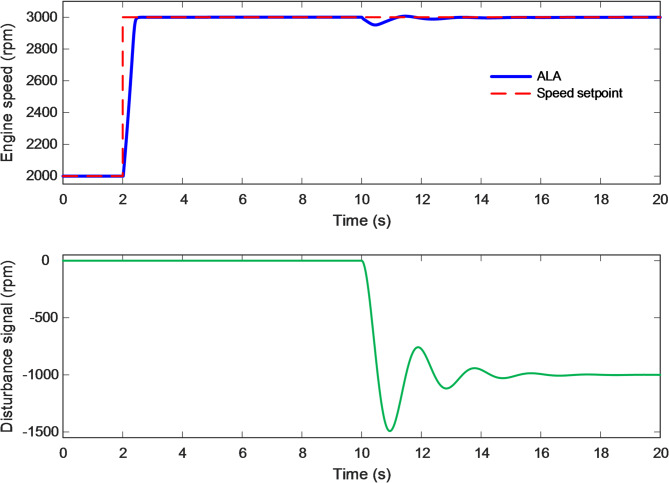
Disturbance rejection efficacy of proposed ALA-based 2-DOF PID controller.

The disturbance-rejection capability of the proposed ALA-optimized nonlinear 2-DOF PID controller was evaluated to assess its robustness under sudden external load perturbations. This analysis provides crucial insight into how effectively the controller can suppress transient deviations and maintain engine-speed stability when the system experiences unexpected torque disturbances. As illustrated in Fig. 12, a negative step disturbance in load torque was introduced at approximately $\:t=10\:s$ to simulate a sudden increase in external resistance acting on the crankshaft. The lower subplot of the figure depicts the disturbance profile, where a sharp negative deflection is observed, corresponding to the abrupt load change. In response, the upper subplot shows the engine-speed behavior under the ALA-based control strategy. The system initially maintained a steady operating speed near 3000 rpm before the disturbance occurred. Upon the introduction of the disturbance, a brief and minor deviation in engine speed was observed, after which the controller promptly restored the speed to its reference value with remarkable smoothness and minimal oscillation. This rapid compensation demonstrates the controller’s strong capacity to counteract torque disturbances through its dual-loop structure. The feedforward path of the 2-DOF PID controller ensured immediate correction by adjusting the throttle opening, while the feedback path refined the response to eliminate residual errors. The adaptive gain tuning achieved via the ALA allowed precise balancing between the proportional and derivative actions, enhancing damping and preventing excessive throttle actuation. Consequently, the system quickly regained steady operation without overshoot or sustained oscillations, indicating that the control effort remained both stable and energy-efficient. Compared with conventional controllers, which often exhibit prolonged recovery or noticeable undershoot during disturbance events, the ALA-tuned system achieved superior resilience. The brief speed dip was confined within an extremely narrow band (less than 0.5% of the nominal speed) highlighting the controller’s precision and robustness. Furthermore, the absence of any oscillatory aftereffects confirms that the nonlinear compensation capability of the optimized 2-DOF PID structure successfully mitigated the nonlinear torque–manifold coupling that typically amplifies disturbance effects in internal combustion engines.

## Conclusions and future works

This study presented a novel ALA–optimized nonlinear 2-DOF PID controller for robust engine speed regulation in spark-ignition systems. By exploiting ALA’s adaptive energy-based search mechanism, the controller achieved a balanced trade-off between rapid transient response and robust steady-state stability. The proposed control architecture was validated through extensive simulation analyses encompassing statistical evaluation, transient and steady-state performance, trajectory tracking, and disturbance rejection. Quantitative results demonstrated the superior performance of the ALA-tuned controller compared to benchmark metaheuristics such as SFOA, PO, COA, and DMO. Across 30 independent trials, ALA consistently delivered the lowest cost-function values and the smallest statistical deviations, verifying its strong convergence stability. Time-domain evaluations confirmed that the ALA-based 2-DOF PID controller achieved faster rise and settling times (0.3114 s and 2.4313 s, respectively), minimal overshoot (0.0027%), and an almost negligible steady-state error. The controller also demonstrated smooth throttle actuation within realistic physical limits and exceptional robustness to both variable speed references and sudden load torque disturbances. The achieved stability and precision highlight the effectiveness of ALA’s adaptive exploration–exploitation transition in fine-tuning control parameters for nonlinear dynamic systems. From an environmental and operational perspective, maintaining precise speed regulation translates to improved fuel efficiency and reduced emissions, as smoother transients minimize throttle excursions and combustion fluctuations. Such control stability can enhance torque smoothness, promote consistent air–fuel mixing, and help after-treatment systems operate within optimal temperature ranges; thereby supporting cleaner and more efficient engine operation.

Despite these advantages, several challenges remain. The computational cost associated with ALA’s iterative population-based process may limit direct deployment in low-power embedded hardware, warranting further optimization or lightweight variants. Additionally, while simulation-based validation confirmed robust performance under dynamic load disturbances, future studies should incorporate parameter uncertainties and perform hardware-in-the-loop (HIL) or real-engine experiments to verify real-time feasibility. The controller’s performance under extreme driving conditions—such as rapid acceleration or abrupt torque transitions—should also be investigated to ensure consistent behavior across all operational regimes.

Future work may focus on enhancing the framework through hybrid optimization strategies, combining ALA with other bio-inspired algorithms to accelerate convergence and reduce computational burden. Incorporating machine learning or reinforcement learning layers could enable online adaptation of the 2-DOF PID gains to continuously optimize performance under evolving operating conditions. Moreover, extending the optimization problem toward multi-objective formulations that simultaneously minimize fuel consumption, emissions, and control effort will align the method with sustainable automotive goals. Evaluations over standardized driving cycles may also be pursued to quantify fuel-economy and emission benefits in realistic conditions. Beyond automotive applications, the adaptability of the proposed ALA-based control paradigm makes it suitable for other nonlinear systems such as hybrid powertrains, renewable-energy converters, precision robotics, and aerospace actuators. The integration of bio-inspired optimization with intelligent control design presented here contributes a generalizable framework for next-generation control systems, capable of delivering high performance, resilience, and efficiency in uncertain and nonlinear environments.

## Data Availability

The datasets used and/or analysed during the current study available from the corresponding author on reasonable request.
